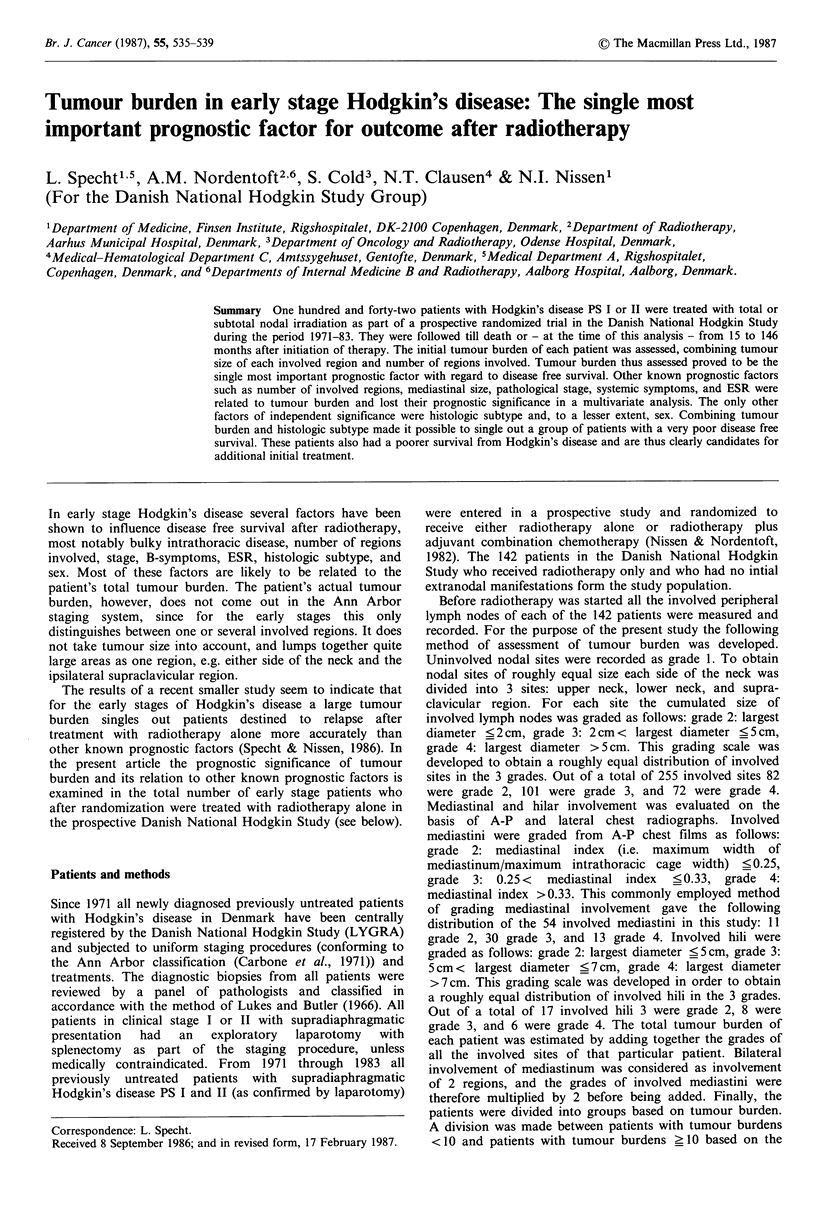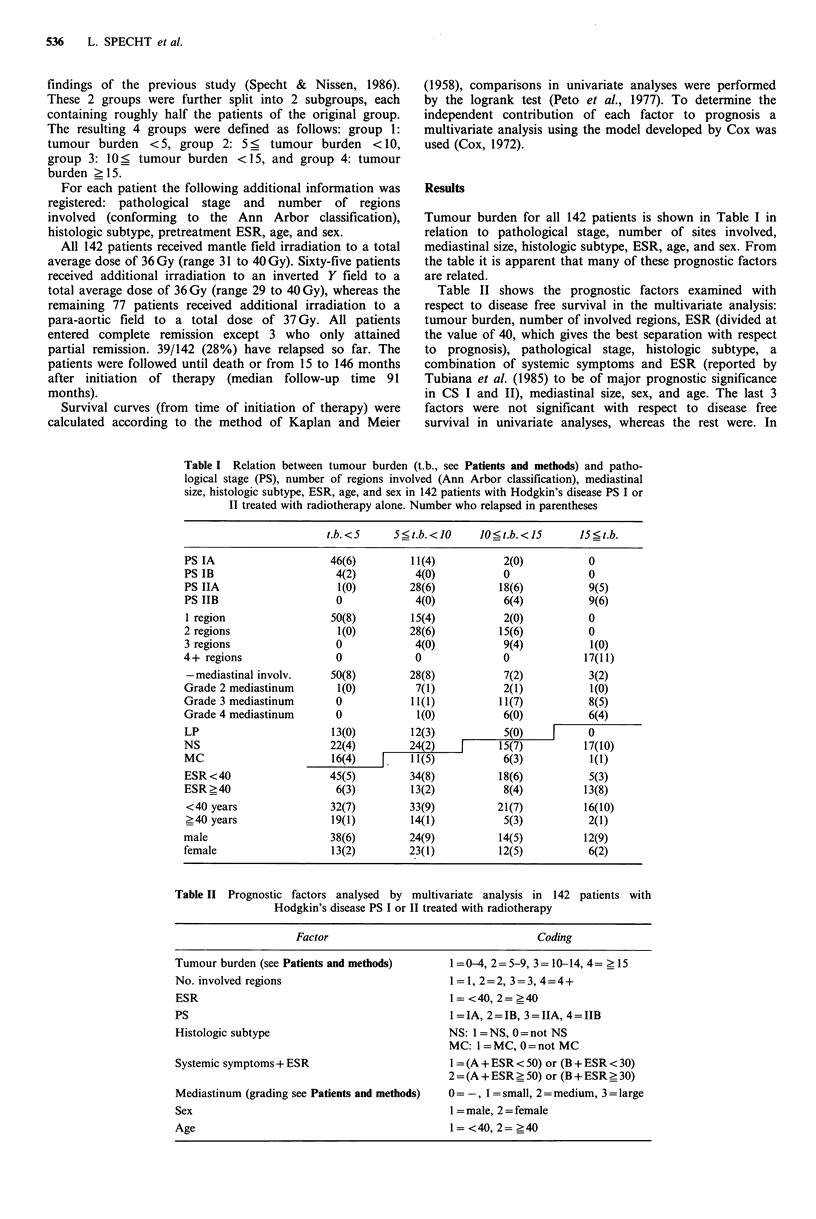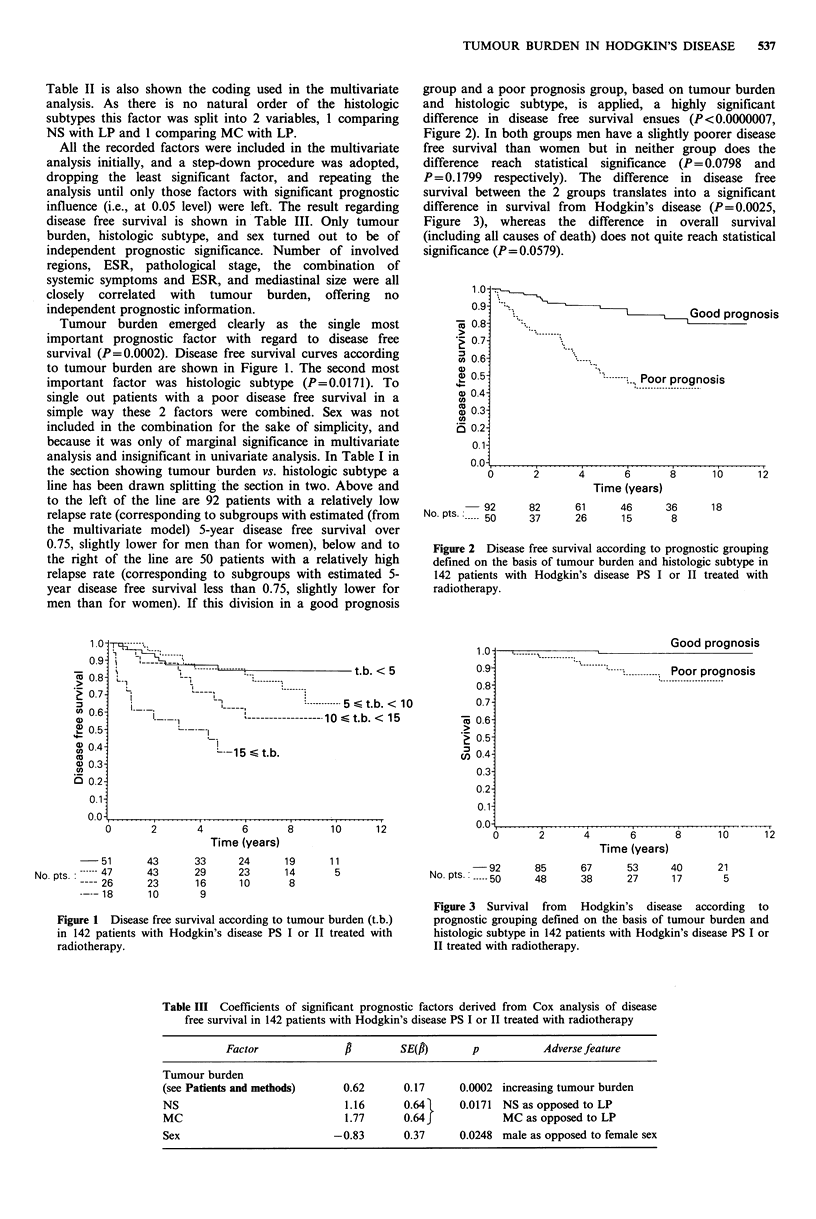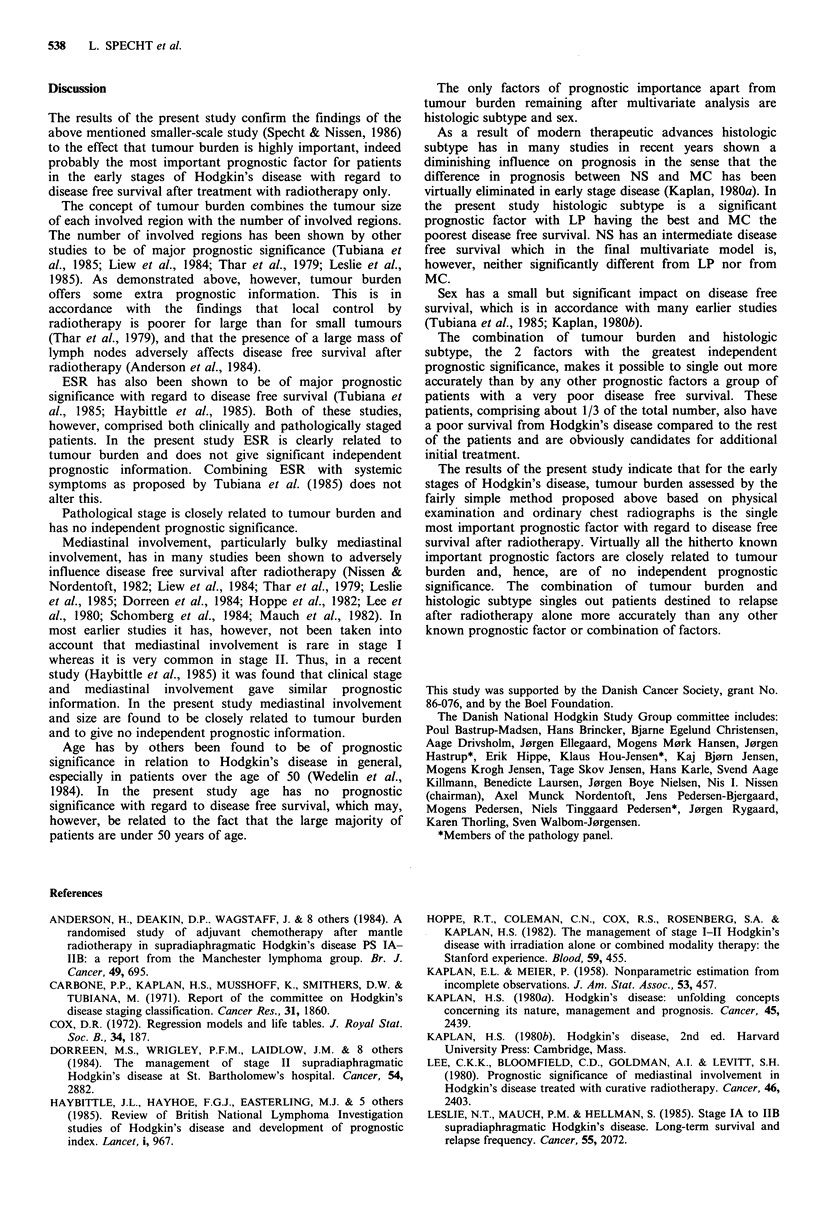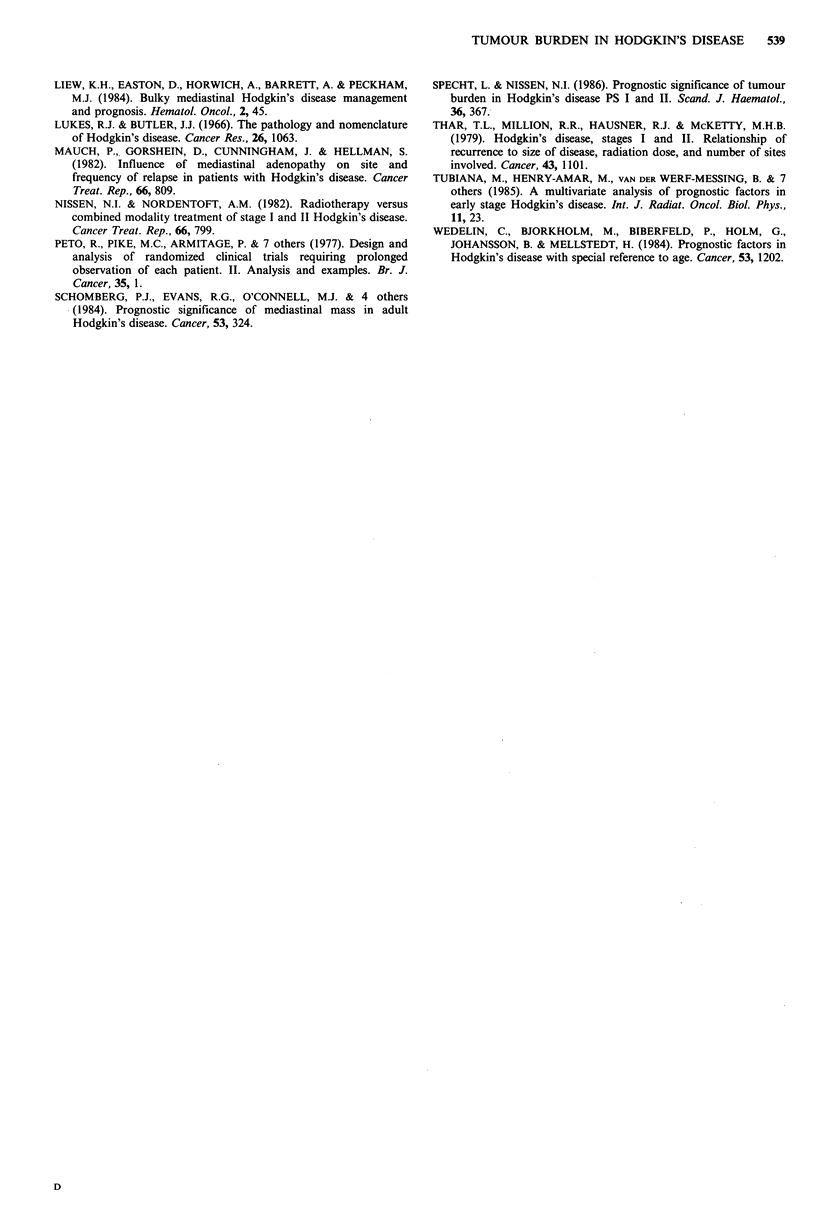# Tumour burden in early stage Hodgkin's disease: the single most important prognostic factor for outcome after radiotherapy.

**DOI:** 10.1038/bjc.1987.109

**Published:** 1987-05

**Authors:** L. Specht, A. M. Nordentoft, S. Cold, N. T. Clausen, N. I. Nissen

## Abstract

One hundred and forty-two patients with Hodgkin's disease PS I or II were treated with total or subtotal nodal irradiation as part of a prospective randomized trial in the Danish National Hodgkin Study during the period 1971-83. They were followed till death or--at the time of this analysis--from 15 to 146 months after initiation of therapy. The initial tumour burden of each patient was assessed, combining tumour size of each involved region and number of regions involved. Tumour burden thus assessed proved to be the single most important prognostic factor with regard to disease free survival. Other known prognostic factors such as number of involved regions, mediastinal size, pathological stage, systemic symptoms, and ESR were related to tumour burden and lost their prognostic significance in a multivariate analysis. The only other factors of independent significance were histologic subtype and, to a lesser extent, sex. Combining tumour burden and histologic subtype made it possible to single out a group of patients with a very poor disease free survival. These patients also had a poorer survival from Hodgkin's disease and thus clearly candidates for additional initial treatment.


Br. J. Cancer (1987), 55, 535–539 © The Macmillan Press Ltd., 1987

### Tumour burden in early stage Hodgkin's disease: The single most important prognostic factor for outcome after radiotherapy

L. Specht[1,5], A.M. Nordentoft[2,6], S. Cold[3], N.T. Clausen[4] & N.I. Nissen[1]
(For the Danish National Hodgkin Study Group)

[1]*Department of Medicine, Finsen Institute, Rigshospitalet, DK-2100 Copenhagen, Denmark,* [2]*Department of Radiotherapy, Aarhus Municipal Hospital, Denmark,* [3]*Department of Oncology and Radiotherapy, Odense Hospital, Denmark,* [4]*Medical–Hematological Department C, Amtssygehuset, Gentofte, Denmark,* [5]*Medical Department A, Rigshospitalet, Copenhagen, Denmark, and* [6]*Departments of Internal Medicine B and Radiotherapy, Aalborg Hospital, Aalborg, Denmark.*

**Summary** One hundred and forty-two patients with Hodgkin's disease PS I or II were treated with total or subtotal nodal irradiation as part of a prospective randomized trial in the Danish National Hodgkin Study during the period 1971–83. They were followed till death or – at the time of this analysis – from 15 to 146 months after initiation of therapy. The initial tumour burden of each patient was assessed, combining tumour size of each involved region and number of regions involved. Tumour burden thus assessed proved to be the single most important prognostic factor with regard to disease free survival. Other known prognostic factors such as number of involved regions, mediastinal size, pathological stage, systemic symptoms, and ESR were related to tumour burden and lost their prognostic significance in a multivariate analysis. The only other factors of independent significance were histologic subtype and, to a lesser extent, sex. Combining tumour burden and histologic subtype made it possible to single out a group of patients with a very poor disease free survival. These patients also had a poorer survival from Hodgkin's disease and are thus clearly candidates for additional initial treatment.

In early stage Hodgkin's disease several factors have been shown to influence disease free survival after radiotherapy, most notably bulky intrathoracic disease, number of regions involved, stage, B-symptoms, ESR, histologic subtype, and sex. Most of these factors are likely to be related to the patient's total tumour burden. The patient's actual tumour burden, however, does not come out in the Ann Arbor staging system, since for the early stages this only distinguishes between one or several involved regions. It does not take tumour size into account, and lumps together quite large areas as one region, e.g. either side of the neck and the ipsilateral supraclavicular region.

The results of a recent smaller study seem to indicate that for the early stages of Hodgkin's disease a large tumour burden singles out patients destined to relapse after treatment with radiotherapy alone more accurately than other known prognostic factors (Specht & Nissen, 1986). In the present article the prognostic significance of tumour burden and its relation to other known prognostic factors is examined in the total number of early stage patients who after randomization were treated with radiotherapy alone in the prospective Danish National Hodgkin Study (see below).

### Patients and methods

Since 1971 all newly diagnosed previously untreated patients with Hodgkin's disease in Denmark have been centrally registered by the Danish National Hodgkin Study (LYGRA) and subjected to uniform staging procedures (conforming to the Ann Arbor classification (Carbone *et al.*, 1971)) and treatments. The diagnostic biopsies from all patients were reviewed by a panel of pathologists and classified in accordance with the method of Lukes and Butler (1966). All patients in clinical stage I or II with supradiaphragmatic presentation had an exploratory laparotomy with splenectomy as part of the staging procedure, unless medically contraindicated. From 1971 through 1983 all previously untreated patients with supradiaphragmatic Hodgkin's disease PS I and II (as confirmed by laparotomy) were entered in a prospective study and randomized to receive either radiotherapy alone or radiotherapy plus adjuvant combination chemotherapy (Nissen & Nordentoft, 1982). The 142 patients in the Danish National Hodgkin Study who received radiotherapy only and who had no intial extranodal manifestations form the study population.

Before radiotherapy was started all the involved peripheral lymph nodes of each of the 142 patients were measured and recorded. For the purpose of the present study the following method of assessment of tumour burden was developed. Uninvolved nodal sites were recorded as grade 1. To obtain nodal sites of roughly equal size each side of the neck was divided into 3 sites: upper neck, lower neck, and supraclavicular region. For each site the cumulated size of involved lymph nodes was graded as follows: grade 2: largest diameter $\leqq 2$ cm, grade 3: $2\,\text{cm} <$ largest diameter $\leqq 5$ cm, grade 4: largest diameter $> 5$ cm. This grading scale was developed to obtain a roughly equal distribution of involved sites in the 3 grades. Out of a total of 255 involved sites 82 were grade 2, 101 were grade 3, and 72 were grade 4. Mediastinal and hilar involvement was evaluated on the basis of A-P and lateral chest radiographs. Involved mediastini were graded from A-P chest films as follows: grade 2: mediastinal index (i.e. maximum width of mediastinum/maximum intrathoracic cage width) $\leqq 0.25$, grade 3: $0.25 <$ mediastinal index $\leqq 0.33$, grade 4: mediastinal index $> 0.33$. This commonly employed method of grading mediastinal involvement gave the following distribution of the 54 involved mediastini in this study: 11 grade 2, 30 grade 3, and 13 grade 4. Involved hili were graded as follows: grade 2: largest diameter $\leqq 5$ cm, grade 3: $5\,\text{cm} <$ largest diameter $\leqq 7$ cm, grade 4: largest diameter $> 7$ cm. This grading scale was developed in order to obtain a roughly equal distribution of involved hili in the 3 grades. Out of a total of 17 involved hili 3 were grade 2, 8 were grade 3, and 6 were grade 4. The total tumour burden of each patient was estimated by adding together the grades of all the involved sites of that particular patient. Bilateral involvement of mediastinum was considered as involvement of 2 regions, and the grades of involved mediastini were therefore multiplied by 2 before being added. Finally, the patients were divided into groups based on tumour burden. A division was made between patients with tumour burdens $< 10$ and patients with tumour burdens $\geqq 10$ based on the

Correspondence: L. Specht.
Received 8 September 1986; and in revised form, 17 February 1987.

findings of the previous study (Specht & Nissen, 1986). These 2 groups were further split into 2 subgroups, each containing roughly half the patients of the original group. The resulting 4 groups were defined as follows: group 1: tumour burden <5, group 2: 5≦ tumour burden <10, group 3: 10≦ tumour burden <15, and group 4: tumour burden ≧15.

For each patient the following additional information was registered: pathological stage and number of regions involved (conforming to the Ann Arbor classification), histologic subtype, pretreatment ESR, age, and sex.

All 142 patients received mantle field irradiation to a total average dose of 36 Gy (range 31 to 40 Gy). Sixty-five patients received additional irradiation to an inverted *Y* field to a total average dose of 36 Gy (range 29 to 40 Gy), whereas the remaining 77 patients received additional irradiation to a para-aortic field to a total dose of 37 Gy. All patients entered complete remission except 3 who only attained partial remission. 39/142 (28%) have relapsed so far. The patients were followed until death or from 15 to 146 months after initiation of therapy (median follow-up time 91 months).

Survival curves (from time of initiation of therapy) were calculated according to the method of Kaplan and Meier (1958), comparisons in univariate analyses were performed by the logrank test (Peto *et al.*, 1977). To determine the independent contribution of each factor to prognosis a multivariate analysis using the model developed by Cox was used (Cox, 1972).

### Results

Tumour burden for all 142 patients is shown in Table I in relation to pathological stage, number of sites involved, mediastinal size, histologic subtype, ESR, age, and sex. From the table it is apparent that many of these prognostic factors are related.

Table II shows the prognostic factors examined with respect to disease free survival in the multivariate analysis: tumour burden, number of involved regions, ESR (divided at the value of 40, which gives the best separation with respect to prognosis), pathological stage, histologic subtype, a combination of systemic symptoms and ESR (reported by Tubiana *et al.* (1985) to be of major prognostic significance in CS I and II), mediastinal size, sex, and age. The last 3 factors were not significant with respect to disease free survival in univariate analyses, whereas the rest were. In

**Table I** Relation between tumour burden (t.b., see **Patients and methods**) and pathological stage (PS), number of regions involved (Ann Arbor classification), mediastinal size, histologic subtype, ESR, age, and sex in 142 patients with Hodgkin's disease PS I or II treated with radiotherapy alone. Number who relapsed in parentheses

| | *t.b.<5* | *5≦t.b.<10* | *10≦t.b.<15* | *15≦t.b.* |
|---|---|---|---|---|
| PS IA | 46(6) | 11(4) | 2(0) | 0 |
| PS IB | 4(2) | 4(0) | 0 | 0 |
| PS IIA | 1(0) | 28(6) | 18(6) | 9(5) |
| PS IIB | 0 | 4(0) | 6(4) | 9(6) |
| 1 region | 50(8) | 15(4) | 2(0) | 0 |
| 2 regions | 1(0) | 28(6) | 15(6) | 0 |
| 3 regions | 0 | 4(0) | 9(4) | 1(0) |
| 4+ regions | 0 | 0 | 0 | 17(11) |
| −mediastinal involv. | 50(8) | 28(8) | 7(2) | 3(2) |
| Grade 2 mediastinum | 1(0) | 7(1) | 2(1) | 1(0) |
| Grade 3 mediastinum | 0 | 11(1) | 11(7) | 8(5) |
| Grade 4 mediastinum | 0 | 1(0) | 6(0) | 6(4) |
| LP | 13(0) | 12(3) | 5(0) | 0 |
| NS | 22(4) | 24(2) | 15(7) | 17(10) |
| MC | 16(4) | 11(5) | 6(3) | 1(1) |
| ESR<40 | 45(5) | 34(8) | 18(6) | 5(3) |
| ESR≧40 | 6(3) | 13(2) | 8(4) | 13(8) |
| <40 years | 32(7) | 33(9) | 21(7) | 16(10) |
| ≧40 years | 19(1) | 14(1) | 5(3) | 2(1) |
| male | 38(6) | 24(9) | 14(5) | 12(9) |
| female | 13(2) | 23(1) | 12(5) | 6(2) |

**Table II** Prognostic factors analysed by multivariate analysis in 142 patients with Hodgkin's disease PS I or II treated with radiotherapy

| *Factor* | *Coding* |
|---|---|
| Tumour burden (see **Patients and methods**) | 1=0–4, 2=5–9, 3=10–14, 4=≧15 |
| No. involved regions | 1=1, 2=2, 3=3, 4=4+ |
| ESR | 1=<40, 2=≧40 |
| PS | 1=IA, 2=IB, 3=IIA, 4=IIB |
| Histologic subtype | NS: 1=NS, 0=not NS<br>MC: 1=MC, 0=not MC |
| Systemic symptoms+ESR | 1=(A+ESR<50) or (B+ESR<30)<br>2=(A+ESR≧50) or (B+ESR≧30) |
| Mediastinum (grading see **Patients and methods**) | 0=−, 1=small, 2=medium, 3=large |
| Sex | 1=male, 2=female |
| Age | 1=<40, 2=≧40 |

Table II is also shown the coding used in the multivariate analysis. As there is no natural order of the histologic subtypes this factor was split into 2 variables, 1 comparing NS with LP and 1 comparing MC with LP.

All the recorded factors were included in the multivariate analysis initially, and a step-down procedure was adopted, dropping the least significant factor, and repeating the analysis until only those factors with significant prognostic influence (i.e., at 0.05 level) were left. The result regarding disease free survival is shown in Table III. Only tumour burden, histologic subtype, and sex turned out to be of independent prognostic significance. Number of involved regions, ESR, pathological stage, the combination of systemic symptoms and ESR, and mediastinal size were all closely correlated with tumour burden, offering no independent prognostic information.

Tumour burden emerged clearly as the single most important prognostic factor with regard to disease free survival ($P = 0.0002$). Disease free survival curves according to tumour burden are shown in Figure 1. The second most important factor was histologic subtype ($P = 0.0171$). To single out patients with a poor disease free survival in a simple way these 2 factors were combined. Sex was not included in the combination for the sake of simplicity, and because it was only of marginal significance in multivariate analysis and insignificant in univariate analysis. In Table I in the section showing tumour burden *vs.* histologic subtype a line has been drawn splitting the section in two. Above and to the left of the line are 92 patients with a relatively low relapse rate (corresponding to subgroups with estimated (from the multivariate model) 5-year disease free survival over 0.75, slightly lower for men than for women), below and to the right of the line are 50 patients with a relatively high relapse rate (corresponding to subgroups with estimated 5-year disease free survival less than 0.75, slightly lower for men than for women). If this division in a good prognosis group and a poor prognosis group, based on tumour burden and histologic subtype, is applied, a highly significant difference in disease free survival ensues ($P < 0.0000007$, Figure 2). In both groups men have a slightly poorer disease free survival than women but in neither group does the difference reach statistical significance ($P = 0.0798$ and $P = 0.1799$ respectively). The difference in disease free survival between the 2 groups translates into a significant difference in survival from Hodgkin's disease ($P = 0.0025$, Figure 3), whereas the difference in overall survival (including all causes of death) does not quite reach statistical significance ($P = 0.0579$).

**Figure 2** Disease free survival according to prognostic grouping defined on the basis of tumour burden and histologic subtype in 142 patients with Hodgkin's disease PS I or II treated with radiotherapy.

**Figure 1** Disease free survival according to tumour burden (t.b.) in 142 patients with Hodgkin's disease PS I or II treated with radiotherapy.

**Figure 3** Survival from Hodgkin's disease according to prognostic grouping defined on the basis of tumour burden and histologic subtype in 142 patients with Hodgkin's disease PS I or II treated with radiotherapy.

**Table III** Coefficients of significant prognostic factors derived from Cox analysis of disease free survival in 142 patients with Hodgkin's disease PS I or II treated with radiotherapy

| *Factor* | *β* | *SE(β)* | *p* | *Adverse feature* |
|---|---|---|---|---|
| Tumour burden (see **Patients and methods**) | 0.62 | 0.17 | 0.0002 | increasing tumour burden |
| NS | 1.16 | 0.64 | 0.0171 | NS as opposed to LP |
| MC | 1.77 | 0.64 | | MC as opposed to LP |
| Sex | −0.83 | 0.37 | 0.0248 | male as opposed to female sex |

### Discussion

The results of the present study confirm the findings of the above mentioned smaller-scale study (Specht & Nissen, 1986) to the effect that tumour burden is highly important, indeed probably the most important prognostic factor for patients in the early stages of Hodgkin's disease with regard to disease free survival after treatment with radiotherapy only.

The concept of tumour burden combines the tumour size of each involved region with the number of involved regions. The number of involved regions has been shown by other studies to be of major prognostic significance (Tubiana *et al.*, 1985; Liew *et al.*, 1984; Thar *et al.*, 1979; Leslie *et al.*, 1985). As demonstrated above, however, tumour burden offers some extra prognostic information. This is in accordance with the findings that local control by radiotherapy is poorer for large than for small tumours (Thar *et al.*, 1979), and that the presence of a large mass of lymph nodes adversely affects disease free survival after radiotherapy (Anderson *et al.*, 1984).

ESR has also been shown to be of major prognostic significance with regard to disease free survival (Tubiana *et al.*, 1985; Haybittle *et al.*, 1985). Both of these studies, however, comprised both clinically and pathologically staged patients. In the present study ESR is clearly related to tumour burden and does not give significant independent prognostic information. Combining ESR with systemic symptoms as proposed by Tubiana *et al.* (1985) does not alter this.

Pathological stage is closely related to tumour burden and has no independent prognostic significance.

Mediastinal involvement, particularly bulky mediastinal involvement, has in many studies been shown to adversely influence disease free survival after radiotherapy (Nissen & Nordentoft, 1982; Liew *et al.*, 1984; Thar *et al.*, 1979; Leslie *et al.*, 1985; Dorreen *et al.*, 1984; Hoppe *et al.*, 1982; Lee *et al.*, 1980; Schomberg *et al.*, 1984; Mauch *et al.*, 1982). In most earlier studies it has, however, not been taken into account that mediastinal involvement is rare in stage I whereas it is very common in stage II. Thus, in a recent study (Haybittle *et al.*, 1985) it was found that clinical stage and mediastinal involvement gave similar prognostic information. In the present study mediastinal involvement and size are found to be closely related to tumour burden and to give no independent prognostic information.

Age has by others been found to be of prognostic significance in relation to Hodgkin's disease in general, especially in patients over the age of 50 (Wedelin *et al.*, 1984). In the present study age has no prognostic significance with regard to disease free survival, which may, however, be related to the fact that the large majority of patients are under 50 years of age.

The only factors of prognostic importance apart from tumour burden remaining after multivariate analysis are histologic subtype and sex.

As a result of modern therapeutic advances histologic subtype has in many studies in recent years shown a diminishing influence on prognosis in the sense that the difference in prognosis between NS and MC has been virtually eliminated in early stage disease (Kaplan, 1980*a*). In the present study histologic subtype is a significant prognostic factor with LP having the best and MC the poorest disease free survival. NS has an intermediate disease free survival which in the final multivariate model is, however, neither significantly different from LP nor from MC.

Sex has a small but significant impact on disease free survival, which is in accordance with many earlier studies (Tubiana *et al.*, 1985; Kaplan, 1980*b*).

The combination of tumour burden and histologic subtype, the 2 factors with the greatest independent prognostic significance, makes it possible to single out more accurately than by any other prognostic factors a group of patients with a very poor disease free survival. These patients, comprising about 1/3 of the total number, also have a poor survival from Hodgkin's disease compared to the rest of the patients and are obviously candidates for additional initial treatment.

The results of the present study indicate that for the early stages of Hodgkin's disease, tumour burden assessed by the fairly simple method proposed above based on physical examination and ordinary chest radiographs is the single most important prognostic factor with regard to disease free survival after radiotherapy. Virtually all the hitherto known important prognostic factors are closely related to tumour burden and, hence, are of no independent prognostic significance. The combination of tumour burden and histologic subtype singles out patients destined to relapse after radiotherapy alone more accurately than any other known prognostic factor or combination of factors.

This study was supported by the Danish Cancer Society, grant No. 86-076, and by the Boel Foundation.

The Danish National Hodgkin Study Group committee includes: Poul Bastrup-Madsen, Hans Brincker, Bjarne Egelund Christensen, Aage Drivsholm, Jørgen Ellegaard, Mogens Mørk Hansen, Jørgen Hastrup*, Erik Hippe, Klaus Hou-Jensen*, Kaj Bjørn Jensen, Mogens Krogh Jensen, Tage Skov Jensen, Hans Karle, Svend Aage Killmann, Benedicte Laursen, Jørgen Boye Nielsen, Nis I. Nissen (chairman), Axel Munck Nordentoft, Jens Pedersen-Bjergaard, Mogens Pedersen, Niels Tinggaard Pedersen*, Jørgen Rygaard, Karen Thorling, Sven Walbom-Jørgensen.

*Members of the pathology panel.

### References

ANDERSON, H., DEAKIN, D.P.. WAGSTAFF, J. & 8 others (1984). A randomised study of adjuvant chemotherapy after mantle radiotherapy in supradiaphragmatic Hodgkin's disease PS IA–IIB: a report from the Manchester lymphoma group. *Br. J. Cancer*, **49**, 695.

CARBONE, P.P., KAPLAN, H.S., MUSSHOFF, K., SMITHERS, D.W. & TUBIANA, M. (1971). Report of the committee on Hodgkin's disease staging classification. *Cancer Res.*, **31**, 1860.

COX, D.R. (1972). Regression models and life tables. *J. Royal Stat. Soc. B.*, **34**, 187.

DORREEN, M.S., WRIGLEY, P.F.M., LAIDLOW, J.M. & 8 others (1984). The management of stage II supradiaphragmatic Hodgkin's disease at St. Bartholomew's hospital. *Cancer*, **54**, 2882.

HAYBITTLE, J.L., HAYHOE, F.G.J., EASTERLING, M.J. & 5 others (1985). Review of British National Lymphoma Investigation studies of Hodgkin's disease and development of prognostic index. *Lancet*, **i**, 967.

HOPPE, R.T., COLEMAN, C.N., COX, R.S., ROSENBERG, S.A. & KAPLAN, H.S. (1982). The management of stage I–II Hodgkin's disease with irradiation alone or combined modality therapy: the Stanford experience. *Blood*, **59**, 455.

KAPLAN, E.L. & MEIER, P. (1958). Nonparametric estimation from incomplete observations. *J. Am. Stat. Assoc.*, **53**, 457.

KAPLAN, H.S. (1980*a*). Hodgkin's disease: unfolding concepts concerning its nature, management and prognosis. *Cancer*, **45**, 2439.

KAPLAN, H.S. (1980*b*). Hodgkin's disease, 2nd ed. Harvard University Press: Cambridge, Mass.

LEE, C.K.K., BLOOMFIELD, C.D., GOLDMAN, A.I. & LEVITT, S.H. (1980). Prognostic significance of mediastinal involvement in Hodgkin's disease treated with curative radiotherapy. *Cancer*, **46**, 2403.

LESLIE, N.T., MAUCH, P.M. & HELLMAN, S. (1985). Stage IA to IIB supradiaphragmatic Hodgkin's disease. Long-term survival and relapse frequency. *Cancer*, **55**, 2072.

LIEW, K.H., EASTON, D., HORWICH, A., BARRETT, A. & PECKHAM, M.J. (1984). Bulky mediastinal Hodgkin's disease management and prognosis. *Hematol. Oncol.*, **2**, 45.

LUKES, R.J. & BUTLER, J.J. (1966). The pathology and nomenclature of Hodgkin's disease. *Cancer Res.*, **26**, 1063.

MAUCH, P., GORSHEIN, D., CUNNINGHAM, J. & HELLMAN, S. (1982). Influence of mediastinal adenopathy on site and frequency of relapse in patients with Hodgkin's disease. *Cancer Treat. Rep.*, **66**, 809.

NISSEN, N.I. & NORDENTOFT, A.M. (1982). Radiotherapy versus combined modality treatment of stage I and II Hodgkin's disease. *Cancer Treat. Rep.*, **66**, 799.

PETO, R., PIKE, M.C., ARMITAGE, P. & 7 others (1977). Design and analysis of randomized clinical trials requiring prolonged observation of each patient. II. Analysis and examples. *Br. J. Cancer*, **35**, 1.

SCHOMBERG, P.J., EVANS, R.G., O'CONNELL, M.J. & 4 others (1984). Prognostic significance of mediastinal mass in adult Hodgkin's disease. *Cancer*, **53**, 324.

SPECHT, L. & NISSEN, N.I. (1986). Prognostic significance of tumour burden in Hodgkin's disease PS I and II. *Scand. J. Haematol.*, **36**, 367.

THAR, T.L., MILLION, R.R., HAUSNER, R.J. & McKETTY, M.H.B. (1979). Hodgkin's disease, stages I and II. Relationship of recurrence to size of disease, radiation dose, and number of sites involved. *Cancer*, **43**, 1101.

TUBIANA, M., HENRY-AMAR, M., VAN DER WERF-MESSING, B. & 7 others (1985). A multivariate analysis of prognostic factors in early stage Hodgkin's disease. *Int. J. Radiat. Oncol. Biol. Phys.*, **11**, 23.

WEDELIN, C., BJORKHOLM, M., BIBERFELD, P., HOLM, G., JOHANSSON, B. & MELLSTEDT, H. (1984). Prognostic factors in Hodgkin's disease with special reference to age. *Cancer*, **53**, 1202.